# Selective Amplification of SPR Biosensor Signal for Recognition of *rpoB* Gene Fragments by Use of Gold Nanoparticles Modified by Thiolated DNA

**DOI:** 10.1186/s11671-017-2031-y

**Published:** 2017-04-04

**Authors:** M. Matsishin, A. Rachkov, A. Lopatynskyi, V. Chegel, A. Soldatkin, A. El’skaya

**Affiliations:** 1 Laboratory of Biomolecular Electronics, Institute of Molecular Biology and Genetics of National Academy of Sciences of Ukraine, 150 Zabolotnogo Str., Kyiv, 03680 Ukraine; 2 Institute of High Technologies, Taras Shevchenko Kyiv National University, 64 Volodymyrska Str., Kyiv, 01003 Ukraine; 3 V.E. Lashkaryov Institute of Semiconductor Physics, NAS of Ukraine, 41, Nauki Ave., Kyiv, 03028 Ukraine

**Keywords:** Gold nanoparticles, SPR, Tuberculosis, DNA hybridization sensor, Signal amplification

## Abstract

An experimental approach for improving the sensitivity of the surface plasmon resonance (SPR) DNA hybridization sensor using gold nanoparticles (GNPs), modified by specific oligonucleotides, was elaborated. An influence of the ionic strength on the aggregation stability of unmodified GNPs and GNPs modified by the thiolated oligonucleotides was investigated by monitoring a value of light extinction at 520 nm that can be considered as a measure of a quantity of the non-aggregated GNPs. While the unmodified GNPs started to aggregate in 0.2 × saline-sodium citrate (SSC), GNPs modified by the negatively charged oligonucleotides were more stable at increasing ionic strength up to 0.5 × SSC. A bioselective element of the SPR DNA hybridization sensor was formed by immobilization on the gold sensor surface of the thiolated oligonucleotides P2, the sequence of which is a fragment of the *rpoB* gene of *Mycobacterium tuberculosis*. The injections into the measuring flow cell of the SPR spectrometer of various concentrations of GNPs modified by the complementary oligonucleotides T2-18m caused the pronounced concentration-dependent sequence-specific sensor responses. The magnitude of the sensor responses was much higher than in the case of the free standing complementary oligonucleotides. According to the obtained experimental data, the usage of GNPs modified by specific oligonucleotides can amplify the sensor response of the SPR DNA hybridization sensor in ~1200 times.

## Background

WHO global report (http://apps.who.int/iris/bitstream/10665/250441/1/9789241565394-eng.pdf) informed about 10.4 million new tuberculosis (TB) cases worldwide in 2015; among them, there were 480,000 new cases of multidrug-resistant TB (MDR-TB). Comparing with the treatment of drug-susceptible tuberculosis, the treatment of MDR-TB is longer and requires more expensive and toxic drugs. *Mycobacterium tuberculosis* develops resistance to rifamycins drug preparations through a mutation in the 81-base pair region of the *rpoB* gene encoding the β-subunit of RNA polymerase [1].

For selective recognition of mutations in this region, the surface plasmon resonance (SPR)-based DNA sensor was developed [2, 3]. The SPR sensor uses an ability of nanoscale gold films to be sensitive to the changes of a dielectric constant of the thin adjacent layer. The DNA hybridization sensors are based on the immobilization of single-stranded oligonucleotide probes onto the sensor surface and the recognition of complementary targets in analyzing samples by their hybridization with the surface-bound probes [4]. If the single-stranded oligonucleotides of specific sequence are appropriately immobilized on this surface (in other words, if the sensor bioselective element is prepared), the complementary sequences from the analyzed DNA sample will hybridize with the immobilized oligonucleotides. This process leads to changes in the refractive index of the specific recognition layer and evokes SPR response in real time [2, 3, 5].

Today, the SPR biosensors represent the most advanced and mature label-free optical biosensor technology. The ability to measure biomolecular interactions directly, in real time and without the use of molecular labels, makes the SPR biosensors a powerful tool for the investigation of such interactions and their kinetic parameters [6]. However, this method, despite its many advantages and facilities, cannot ensure the detection of DNA sequences in subnanomolar concentrations without using molecular labels [7].

A sandwich assay can be used to improve the detection limit of the SPR method. This approach consists of two consecutive steps: (1) identification of a target DNA by the selective hybridization with DNA probes immobilized on the sensor surface and (2) amplification of the sensor response by the additional hybridization step between a target DNA and so-called detection probes attached to an amplification agent. A key to the success of this signal amplification approach is an appropriate choice of the amplification agent. In the case of SPR method, a molecular label can be a big macromolecule or a nanoobject, for example a gold nanoparticle.

Gold nanoparticles (GNPs) possess many attractive features: they have a quite low price (1 L of 10 nM GNPs of 13 nm diameter costs ∼$3.7) [8], they are not toxic, and they can be easily modified by various chemicals or biomacromolecules [9]. Therefore, the choice of GNPs fits well for the development of technologically simple, flexible, and cheap method of amplification of the SPR sensor signal.

The main purpose of this work was to elaborate an experimental approach for improving the sensitivity of the SPR DNA hybridization sensor using the GNPs modified by specific oligonucleotides and to evaluate a magnitude of the amplification of the sensor response, which can be achieved by using the elaborated approach.

## Methods

### Reagents

Urea, KH_2_PO_4_, and 6-mercapto-1-hexanol were obtained from Fluka (Buchs, Switzerland); all other reagents were of analytical grade. All solutions were made with deionized Milli-Q water.

For immobilization on a gold sensor surface, the single-stranded oligonucleotide P2 functionalized at the 5′-end with a thiol group through hexamethylene spacer (HS-(CH_2_)_6_-ACC CAC AAG CGC CGA CTG TTG) was used. Its sequence represents the fragment of the *rpoB* gene of *M. tuberculosis*, the mutations of which lead to the drug resistance of the bacteria.

For selective hybridization, the thiolated oligonucleotide T2-18m (HS-(CH_2_)_6_-CAA CAG TCG GCG CTT GTG) and the unmodified oligonucleotide T2 (CAA CAG TCG GCG CTT GTG GGT) were applied. It should be noted that these two oligonucleotides possess a homological sequence; T2-18m is shorter than T2 by three nucleotides from the 3′-end. Both oligonucleotides should hybridize with the complementary oligonucleotide P2 in a very similar manner.

For testing selectivity of the sensor response, the thiolated oligonucleotide mod-Ph (HS-(CH_2_)_6_-GCTGAAGGGCTTTTGAACTCTGCT), which is non-complementary to P2, was used.

All oligonucleotides were obtained from Metabion International AG (Germany). The choice of the length and nucleotide sequence of the oligonucleotides was described elsewhere [5].

### Immobilization of Thiolated Oligonucleotides on the Sensor Surface

To investigate the processes of oligonucleotide immobilization and hybridization, we used the two-channel SPR spectrometer “Plasmon SPR-6.” This computer-controlled optoelectronic device in the Kretchmann’s optical configuration was developed at V.E. Lashkaryov Institute of Semiconductor Physics of National Academy of Sciences of Ukraine. The 45-nm-thick gold layer on a glass plate serves as a sensor surface. A specific recognition layer on the sensor surface (bioselective element) is prepared as follows. Prior to modification, gold surface of the glass plate is cleaned with freshly prepared piranha solution (3:1 mixture of concentrated H_2_SO_4_ and 30% H_2_O_2_; warning: *piranha solution reacts violently with organic compounds and must be handled with extreme care*) at room temperature for 2 min, then rinsed thoroughly with distilled and deionized water, and dried in the air. The cleaned plate is mounted on the spectrometer prism using immersion liquid. The flow rate (usually 40 μl min^−1^) is controlled by the peristaltic pump “Ismatec.” For immobilization of the thiolated probe, 120 μl of 1 μM P2 in 0.5 M KH_2_PO_4_ (pH 3.8) is injected into the measuring flow cell and exposed for 1 h. After that, the sites on the gold surface free from immobilized P2 were blocked by 1 mM aqueous solution of 6-mercapto-1-hexanol [10].

### Hybridization Experiments

At the beginning of the hybridization experiment, the measuring flow cell is thoroughly washed by the running buffer solution, for example, 2× saline-sodium citrate (SSC) (30 mM sodium citrate, 300 mM NaCl, pH 7) to obtain a stable sensor signal. At the next step, 120 μl of target solutions of various concentration in the running buffer solution is injected into the measuring flow cell and exposed for 10 min. To distinguish an actual sensor response caused by the interactions between the target and the immobilized probe from that caused by the occasional fluctuations of medium refractive index, it is necessary to wash the flow cell before and after each sample by the same buffer solution, and only then to determine a value of the SPR response.

### Synthesis of GNPs

The preparation of GNPs was performed through the reduction of tetrachloroaurate ions (AuCl_4_^−^) by boiling in an aqueous sodium citrate solution [11]. First, 20 ml of 1 mM HAuCl_4_ was heated to a boil with constant stirring and with reflux. Then, 2 ml of 1% sodium citrate was added to it. Heating and stirring were continued for 10 min. Gold ions were reduced gradually, and the color of the solution became saturated red. The solution was cooled to room temperature. GNPs obtained using this method appear as almost monodispersed spherical structures with a size of about 10–15 nm, which are stabilized by weakly bound citrate ions. GNPs are characterized by the plasmonic absorption band at approximately 520 nm [12–14].

### Modification of GNPs

For reliable immobilization of thiolated oligonucleotides onto GNPs, an ability of their SH-groups to form covalent bonds with gold surface was used. To determine a level of the oligonucleotide immobilization on the surface of GNPs, the procedure based on the property of SYBR Green II to drastically increase its fluorescence in the presence of a single-stranded oligonucleotide was applied. Namely, a level of fluorescence of SYBR Green II (proportional to the oligonucleotide concentration) in solutions before and after immobilization (initial concentration and concentration of unbound oligonucleotides) was measured. The difference allows estimating a number of attached oligonucleotides.

GNPs modification by the thiolated oligonucleotides was carried out by incubation of GNPs with oligonucleotides during one day in 0.1 × SSC buffer solution. After that, nanoparticles were separated from the unbound oligonucleotides by centrifugation at 10,000 rpm. The supernatant was used to determine a level of the oligonucleotide immobilization on the surface of GNPs by SynergyHT (BioTek) Microplate Reader, and the precipitate was analyzed by UV-vis spectra analysis using spectrophotometer Nanodrop2000.

## Results and Discussion

In order to elaborate an experimental approach for improving the sensitivity of the SPR DNA hybridization sensor using GNPs modified by specific oligonucleotides, it was necessary to find compromising conditions for two simultaneous phenomena: an aggregation stability of GNPs and a quite efficient DNA hybridization. It is well known that an efficiency of DNA hybridization directly depends on the ionic strength of medium. For example, in our previous works, DNA hybridization on the SPR sensor surface was performed in 1 × SSC [5] or in 2 × SSC [2] buffer solutions, the ionic strength of which exceeded 150 or 300 mM, respectively. Under such conditions, an equilibrium between attractive hydrogen bonds and repulsive electrostatic forces in dispersion of GNPs is disturbed and electrolyte-induced aggregation occurs. The dispersed GNPs exhibit only a single peak at ~520 nm, while linked particle pairs (or larger aggregates) show two light extinction maxima. As the interparticle spacing decreases, the first peak becomes weaker, while the second peak intensifies and shifts to longer wavelengths [12–14].

Therefore, an influence of the ionic strength (various multiplicity of SSC buffer solution) on the aggregation stability of GNPs was investigated by monitoring a value of extinction at 520 nm that can be considered as a measure of a quantity of the non-aggregated GNPs. We compared the unmodified GNPs and two preparations of GNPs modified by the thiolated oligonucleotides T2-18m—one with the average value of 13 DNA strands per nanoparticle (it was obtained by incubation of 2 nM GNPs and 200 nM T2-18m during one day in 0.1 × SSC buffer solution) and the other one with the average number of 22 DNA strands per nanoparticle (it was obtained by incubation of 5 nM GNPs and 1 μM T2-18m during one day in 0.1 × SSC buffer solution).

**Fig. 1:**
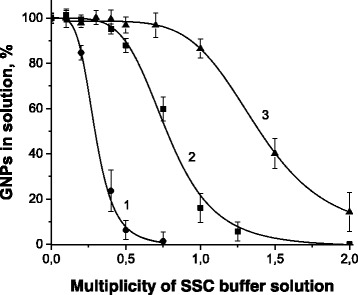
The influence of ionic strength (multiplicity of SSC buffer solution) on the level of light extinction at 520 nm of the unmodified GNPs (*1*) and two preparations of GNPs modified by the thiolated oligonucleotides T2-18m—one sample with the average number of 13 DNA strands per particle (*2*) and the other with the average number of 22 DNA strands per particle (*3*)

The obtained results confirmed the validity of the earlier described findings that the fate of modified GNPs depends on a charge which is carried by the modifying compounds and the negatively charged compounds increase the stability of nanoparticle dispersion [15]. Indeed, while the unmodified GNPs started to aggregate in 0.2 × SSC, almost complete disappearance of the light extinction at 520 nm in 0.5 × SSC can be observed (Fig. 1 (curve 1)); GNPs modified by the negatively charged oligonucleotides are more stable at increasing ionic strength (Fig. 1 (curves 2 and 3)). Herewith, GNPs with a higher surface density of the immobilized oligonucleotides demonstrated a higher stability. Their level of light extinction at 520 nm remained unchanged up to 0.5 × SSC. The obtained results are rather encouraging because this concentration of the buffer solution is high enough to be appropriate for DNA hybridization. Moreover, 0.5 × SSC buffer solution provides rather stringent hybridization conditions and makes the process of hybridization highly selective [3, 16].

A bioselective element of the SPR DNA hybridization sensor was formed by immobilization of the thiolated oligonucleotides P2 on the gold sensor surface; free sites of the surface were blocked by 6-Mercapto-1-hexanol to reduce a nonspecific adsorption. The 0.5 × SSC buffer solution was used as a running buffer solution for continuous washing the measuring flow cell of the SPR spectrometer and for preparation of all investigated samples.

**Fig. 2:**
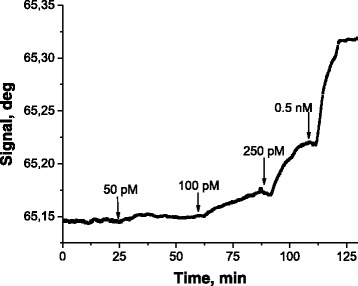
The SPR sensogram obtained at the consecutive injections of various concentration of GNPs modified by the thiolated oligonucleotides T2-18m with the average number of 22 DNA strands per particle

Figure 2 shows the sensor response on the consecutive injections of various concentration of GNPs modified by the thiolated oligonucleotides T2-18m with the average number of 22 DNA strands per particle. Statistically reliable sensor response (sensor-to-noise ratio >3) can be seen starting with 100-picomolar concentration of GNPs. This chart represents a concentration-dependent response throughout the whole range of the studied concentrations.

**Fig. 3:**
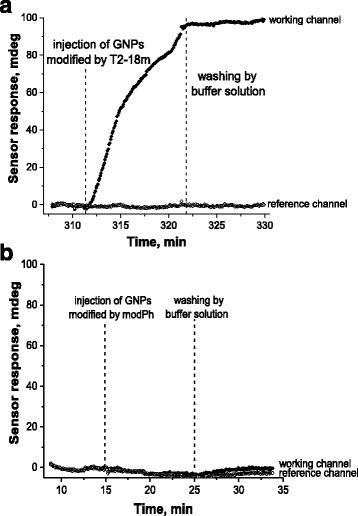
The SPR sensorgrams, which demonstrate the SPR DNA hybridization sensor response on the injections of 0.5 nM GNPs modified by the complementary oligonucleotides T2-18m (**a**) and 0.5 nM GNPs modified by the non-complementary oligonucleotides mod-Ph (**b**). Reference channel was washed by the running buffer solution only

To check how selective the obtained sensor response is, we compared obtained for GNPs modified by the oligonucleotides T2-18m with that for GNPs modified by the non-complementary oligonucleotides mod-Ph (Fig. 3). Both preparations of GNPs were of the same concentration of nanoparticles (according to their light extinction at 520 nm) and have rather close levels of the surface density of the immobilized oligonucleotides.

The injection into the measuring flow cell of 0.5 nM GNPs modified by T2-18m gave a response of approximately 100 mdeg, while the injection of 0.5 nM GNPs modified by mod-Ph gave a response less than 1 mdeg. The last one is comparable with the noise level and cannot be considered as a specific biosensor response. It should be noted that 0.5 nM GNPs modified by T2-18m gave a three times bigger sensor response than that after the injection of 400 nM of the free standing complementary oligonucleotides T2 (this T2 concentration causes the saturation of the sensor response). It is obvious that so big difference in the sensor responses on the injections of GNPs modified by the complementary or the non-complementary oligonucleotides (Fig. 3) has been caused by selective hybridization of the first one and by accumulation of GNPs near the sensor surface.

**Fig. 4:**
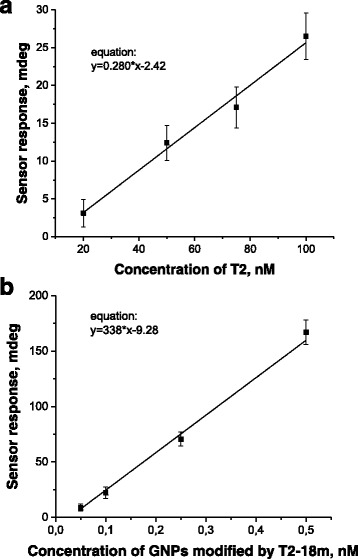
The linear ranges of the calibration curves of the SPR DNA hybridization sensor response on the injections of the complementary free standing oligonucleotides T2 (**a**) or on the injections of GNPs modified by the complementary oligonucleotides T2-18m (**b**)

In order to determine a magnitude of the amplification, which can be achieved by using the elaborated approach, we have constructed two calibration curves (within their linear ranges). In both cases, the bioselective element of the SPR biosensor was based on the oligonucleotide P2. The first curve shows the SPR biosensor response on the free standing oligonucleotides T2 (complementary to P2) (Fig. 4a), and another one the SPR biosensor response on GNPs modified by oligonucleotides T2-18 m (also complementary to P2) (Fig. 4b). A ratio of the slopes of these two lines can be considered as a magnitude of amplification achieved by using the elaborated approach. Taking into consideration the obtained slope values 338 and 0.28 mdeg/nM, one can calculate that the elaborated approach can amplify a response of the SPR DNA hybridization sensor in ~1200 times.

## Conclusions

The elaborated experimental approach using GNPs modified by specific oligonucleotides showed the significant improvement of the sensitivity of the SPR DNA hybridization sensor. It can provide maximal amplification rate up to 1200 times. In the case of sandwich assay, a level of hybridization of specific oligonucleotides attached to GNPs will be restricted by a number of analyzed DNA targets, which were already linked to the captured oligonucleotides immobilized on the sensor surface during the first step of analysis. Nevertheless, the elaborated experimental approach could provide a significant increase in sensor response and an essential decrease in detection limit.

## References

[1] Espinal MA, Kim SJ, Suarez PG (2000). Standard short-course chemotherapy for drug-resistant tuberculosis. JAMA.

[2] Rachkov A, Patskovsky S, Soldatkin A, Meunier M (2011). Surface plasmon resonance detection of oligonucleotide sequences of the rpoB genes of Mycobacterium tuberculosis. Talanta.

[3] Rachkov A, Patskovsky S, Soldatkin A, Meunier M (2013). Discrimination of single base mismatched oligonucleotides related to the rpoB gene of Mycobacterium tuberculosis using a surface plasmon resonance biosensor. Biotechnol Appl Biochem.

[4] Teles F, Fonseca L (2008). Trends in DNA biosensors. Talanta.

[5] Rachkov A, Holodova Y, Ushenin Y (2009). Development of bioselective element of SPR spectrometer for monitoring of oligonucleotide interactions and comparison with thermodynamic calculations. Sens Lett.

[6] Šípová H, Homola J (2013). Surface plasmon resonance sensing of nucleic acids: a review. Anal Chim Acta.

[7] Fan X, White IM, Shopova SI (2008). Sensitive optical biosensors for unlabeled targets: a review. Anal Chim Acta.

[8] McFarland AD, Haynes CL, Mirkin CA (2004). Color My Nanoworld. J Chem Educ.

[9] Merkoçi A (2010). Nanoparticles-based strategies for DNA, protein and cell sensors. Biosens Bioelectron.

[10] Herne TM, Tarlov MJ (1997). Characterization of DNA probes immobilized on gold surfaces. J Am Chem Soc.

[11] Turkevich J, Stevenson PC, Hillier J (1951). A study of the nucleation and growth processes in the synthesis of colloidal gold. Discuss Faraday Soc.

[12] Ghosh SK, Pal T (2007). Interparticle coupling effect on the surface plasmon resonance of gold nanoparticles: from theory to applications. Chem Rev.

[13] Xiao Q, Gao H, Lu C, Yuan Q (2012). Gold nanoparticle-based optical probes for sensing aminothiols. TrAC Trends Anal Chem.

[14] Zhao W, Brook MA, Li Y (2008). Design of gold nanoparticle-based colorimetric biosensing assays. ChemBioChem.

[15] Chegel V, Rachkov O, Lopatynskyi A (2012). Gold nanoparticles aggregation: drastic effect of cooperative functionalities in a single molecular conjugate. J Phys Chem C.

[16] Matsishin MJ, Ushenin IV, Rachkov AE, Solatkin AP (2016). SPR detection and discrimination of the oligonucleotides related to the normal and the hybrid bcr-abl genes by two stringency control strategies. Nanoscale Res Lett.

